# Supplementary material for: BRCA2 C-Terminal RAD51-Binding Domain Confers Resistance to DNA-Damaging Agents
Source: Int J Mol Sci. 2022 Apr 6;23(7):4060. doi: 10.3390/ijms23074060 (PMC9000072; doi:10.3390/ijms23074060)
Supplement: Supplementary file 1 [file ijms-23-04060-s001.zip › ijms-1642325-supplementary.pdf]

Supplementary Material

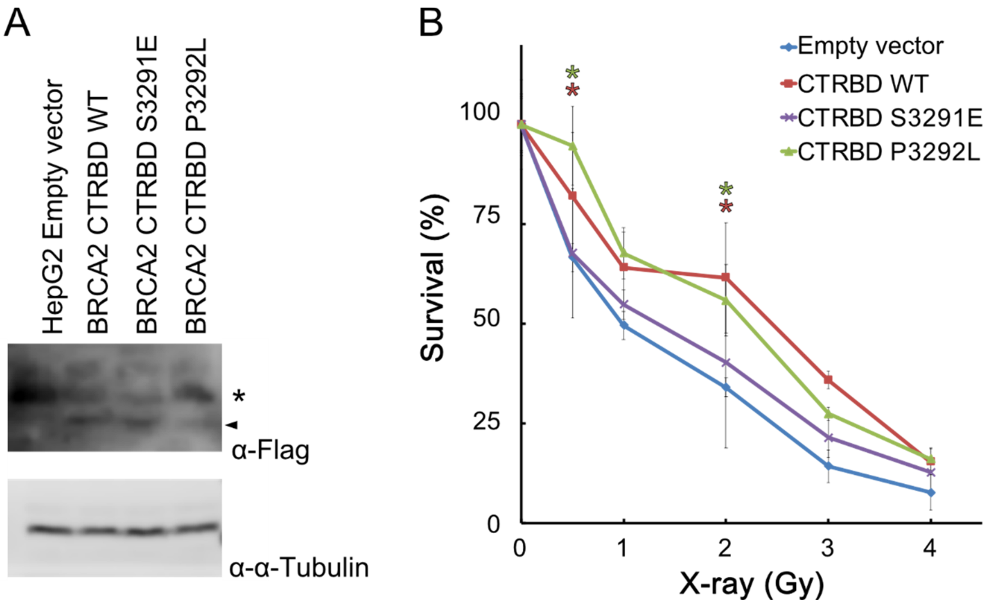

Figure S1. C-terminal RAD51-binding domain (CTRBD) also confers resistance to X-ray irradiation and mitomycin C (MMC) treatment in HepG2 cells. (**A**) Expression of exogenous FLAG-HA-fused BRCA2 CTRBD (3260–3331 aa) and its mutants (S3291E and P3292L) was evaluated by western blotting, using an anti-FLAG antibody. Asterisks indicate nonspecific bands. (**B**) A clonogenic survival assay of FLAG-HA-fused BRCA2 CTRBD-expressing cells treated with X-ray irradiation is shown. Results are presented as the mean at each X-ray or MMC dose, and the error bars indicate the standard deviation (n = 3). Significance was examined by F-test, followed by Student's t-test with Holm's correction. Asterisks indicate significant differences between empty vector-transduced HeLa cells and FLAG-HA-fused wild-type or P3292L-mutated BRCA2 CTRBD-expressing cells (*: $p < 0.05$).
